# The optimal puncture time point of prolonged occlusion flow-mediated dilatation in radial artery catheterization: a prospective observational study

**DOI:** 10.1038/s41598-023-49122-0

**Published:** 2023-12-08

**Authors:** Wensheng Qi, Haichun Ma, Xuehan Wu, Kun Wei, Yanhui Li

**Affiliations:** 1 Department of Anesthesiology, First Hospital of Jilin University, 1 Xinmin Street, Chaoyang District, Changchun, China; 2 Department of Rehabilitation, Second Hospital of Shandong University, Jinan, China

**Keywords:** Physiology, Cardiology

## Abstract

Previous studies have demonstrated prolonged occlusion flow-mediated dilatation (PO-FMD) could reduce cannulation failure rates and decrease radial artery pulsation loss during trans-radial coronary angiography. However, the time and degree of radial artery dilatation induced after PO-FMD were unclear. This study aimed to evaluate the degree and duration of the radial artery dilation after PO-FMD, and the time point at which the radial artery diameter is expanded to the maximum. This was a prospective observational study. According to the Chinese guideline on the primary prevention of cardiovascular diseases, 142 patients awaking from general anesthesia were divided into two groups: low-risk (LR) group and high-risk (HR) group. Firstly, the baseline radial artery diameter was measured on the left wrist using ultrasound in both groups. Subsequently, the radial artery diameters were obtained continuously at the same location for 5 min after PO-FMD. The baseline radial artery diameter, the maximum radial artery diameter, and the duration of radial artery dilation in the two groups were recorded. The time point at which the radial artery diameter is expanded to the maximum in the LR group and HR group was 26.49 ± 11.69 s and 46.27 ± 12.03 s, respectively (*P* < 0.01). The time of radial artery dilation and the percentage changes in arterial diameter in HR group were significantly lower than LR group (duration time: mean [mean ± standard]: 136.65 ± 31.55 s vs. 168.98 ± 33.27 s; percentage changes: median [interquartile range] 10.5 [8.6, 12.9] % vs. 15.2 [12.4, 19.0] %). In this study, the optimal puncture time point of PO-FMD in the LR group was 26 s, and in the HR group was 46 s. It would be helpful to guide the time point in radial artery catheterization after PO-FMD.

*Chinese Clinical Trial Registry identifier*: ChiCTR2200066214.

## Introduction

Monitoring arterial blood pressure continuously is one of the most important approaches for maintaining hemodynamic stability during the perioperative period. Radial artery catheterization is widely accepted as an easy and safe technique for continuous hemodynamic monitoring and repeated arterial blood gas analysis to maintain hemodynamic stability and the water-electrolyte acid–base balance^1^. Radial artery catheterization, particularly in patients with obesity, severe burns, and shock, often is challenging and may require several attempts^2^. Additionally, repeated attempts at catheterization may cause clinical complications and patient discomfort. Therefore, it is essential to improve the first-attempt success rate.

Several strategies can be used to increase access success through dilating the radial artery inner diameter, including acetylcholine infusion, sublingual nitroglycerin, subcutaneous nitroglycerin, warmed hand, and flow-mediated dilatation (FMD)^3–5^. FMD is a normal physiological process whereby the arteries dilate in response to an increase in blood flow. It occurs when endothelial cells respond to vascular wall shear stress by increasing their synthesis of vasodilatory substances^6^. In clinical, as an assessment of endothelial function, FMD in vivo has gained widespread acceptance. If endothelial dysfunction can be identified prior to the development of atherosclerosis, interventions to prevent the disease may be used. The time and magnitude of this nitric oxide-dependent dilatation are significantly diminished in people with atherosclerosis. Furthermore, with the occlusion times prolonged, the time and magnitude of vasodilatation increased even in people with atherosclerosis^5^. As compared with other methods which could improve radial artery puncture’s success rate, PO-FMD is easy, cheap, safe, and requires little specialized training or equipment.

FMD is rarely reported as an adjunctive technique despite the application for assessing coronary and peripheral artery disease risk^7–9^. Previous studies have demonstrated that PO-FMD could reduce catheterization failure rates, decrease puncture attempts, and decrease radial artery pulsation loss during trans-radial coronary angiography^10^. In their study, cardiologists performing angiography were blinded to ultrasound measurements of radial artery size. And they performed arterial puncture immediately after PO-FMD. Therefore, we examined the time and degree of radial artery dilatation induced after PO-FMD in CVD low-risk patients and CVD high-risk patients. By quantifying the degree and time of radial artery dilatation, this will make clinical implementation of the adjunctive technology much more feasible. The main aim of the present study was helpful to guide the time point in radial artery catheterization after PO-FMD.

## Methods

### Patient population

**Figure 1:**
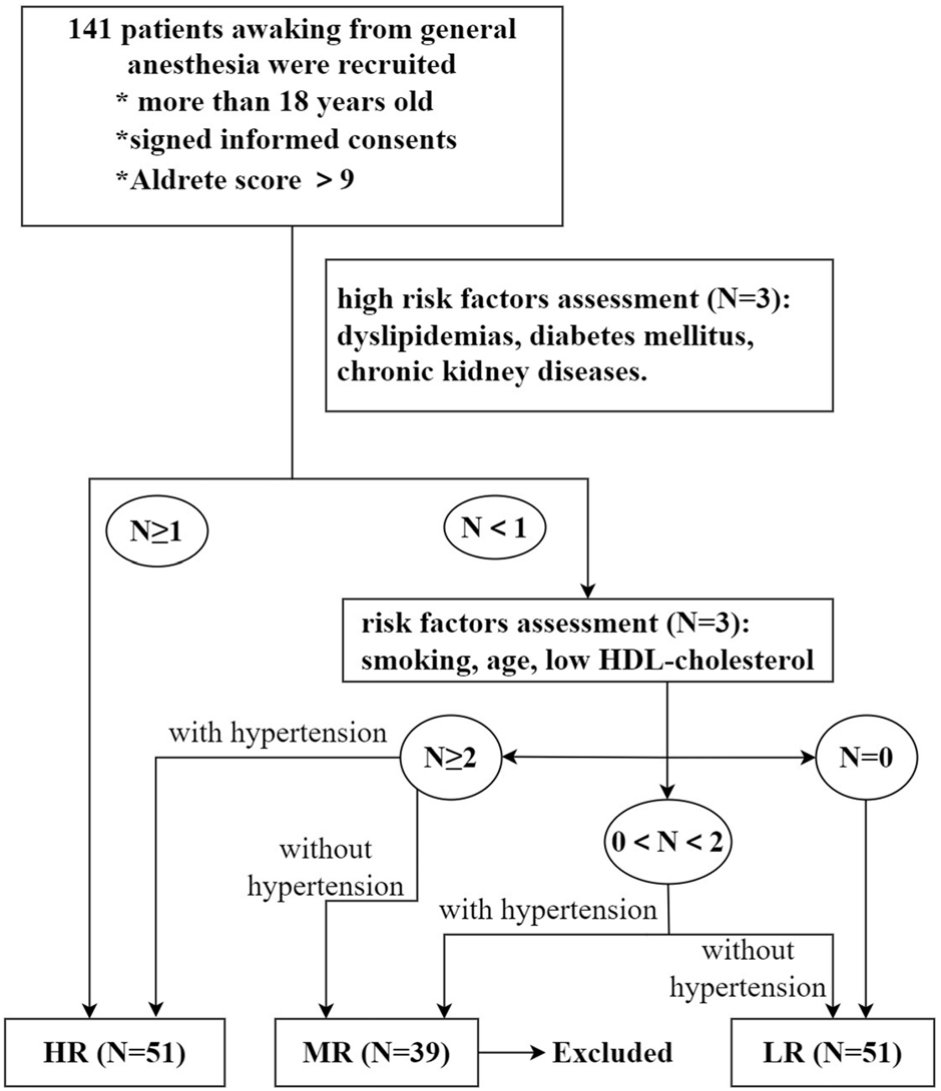
Show a flow diagram describing the risk stratification process for all patients. LR, low-risk group; MR, medium-risk group; HR, high-risk group.

This was a prospective, single-center, open-label, nonrandomized pilot study approved by the First Hospital of Jilin University Ethics Committee (22K068001), Changchun, China (chairperson: Dr Guo Di) on 10 October 2022. Prior to screening procedures, written informed consent was obtained from each patient. The study was conducted with the guidelines provided by the World Medical Association Declaration of Helsinki on Ethical Principles for Medical Research Involving Humans, and we confirmed that all experiments were performed in accordance with relevant guidelines and regulations. Recovering patients from general anesthesia in the First Hospital of Jilin University were recruited for the study. Patients who had a severe peripheral vascular disease or who had coagulation function abnormalities or who had instability hemodynamics were excluded from the study. Also, patients who had been assessed as medium-risk were excluded from the study. One hundred and forty-one patients (62 males, 79 females) were recruited in total. Following the protocols designed by the Chinese guideline on the primary prevention of cardiovascular diseases^11^, all patients had their risk factors evaluated. A flow chart detailing the study selection process is shown in Fig. 1.

### Risk factor assessment

The high-risk factors (HRFs) assessed were age, dyslipidemias, diabetes mellitus, and chronic kidney diseases. HRF1: Diabetes patients with an age of onset greater than 40 years. Diabetes mellitus was defined as the prior medical history of diabetes mellitus or fasting plasma glucose concentration ≥ 7.0 mmol/L. HRF2: Dyslipidemia was defined by having one or more of the following characteristics: low-density lipoprotein ≥ 4.9 mmol/L or total cholesterol ≥ 7.2 mmol/L, or currently use of statins. HRF3: Patients with chronic kidney diseases (CKD) III–IV stage. CKD stage III was defined as glomerular filtration rate (GFR) 30–59 mL/min/1.73 m^2^; stage IV, GFR = 15–29 ml/min/1.73 m^2^. The other risk factors assessed were smoking, age, and low HDL cholesterol. RF1: A self-reported history of smoking and smoking habits is collected. RF2: Age ≥ 45/55 years (male/female). RF3: The low level of HDL-cholesterol was defined as high-density lipoprotein cholesterol of less than 1.0 mmol/L. Hypertension is an independent risk factor defined as systolic blood pressure ≥ 140 mmHg or diastolic blood pressure ≥ 90 mmHg, or current treatment with antihypertensive agents.

The high-risk (HR) group was defined as patients with one or more high-risk factors or patients who had hypertension with two or more risk factors. Patients without high-risk factors were divided into two groups: those without hypertension with fewer than two risk factors or patients who had hypertension without any risk factors were classified as low-risk (LR) group, and the other patients were classified as medium-risk (MR) group.

### Protocol

**Figure 2:**
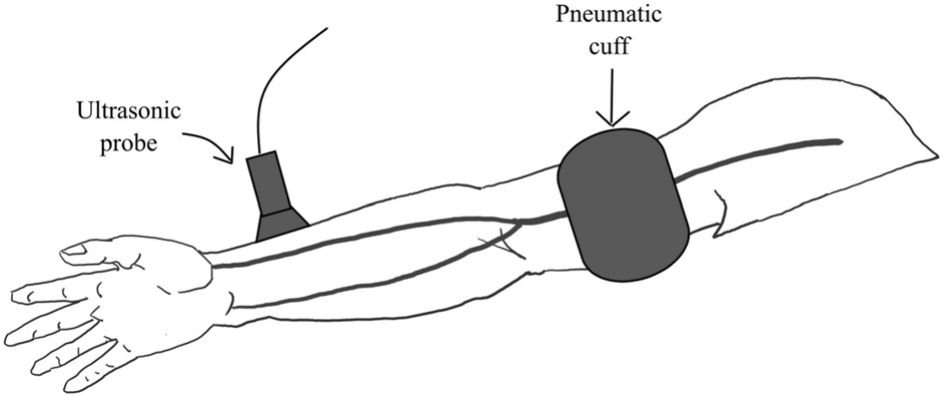
Experimental step: Inflation of an upper arm cuff to a pressure 30 mmHg higher than the patient’s baseline systolic blood pressure for 10 min, after which it was deflated and the radial artery diameter was measured using ultrasound.

After arrival in the recovery room, the patients were monitored for non-invasive blood pressure, pulse oxygen saturation, heart rate, and continuous electrocardiogram. Patients displaying an Aldrete score higher than nine are possible cases. A non-invasive automatic device was used to measure baseline systolic blood pressure (averaged over three measurements) at 3-min intervals. The patient’s left arm was immobilized comfortably in an extended position so that it could be accessed consistently to the radial artery for imaging. The operator used a high-resolution Doppler ultrasound (Wisonic Navi, Shenzhen, China) with a 10 MHz linear array transducer to obtain radial artery diameter images. The position and shape of the radial artery were explored from the site 2 cm proximal to the radial styloid process of the radial bone to the proximal end. Ensuring the radial artery interface with the largest lumen diameter and the clearest image, and recorded continuously by ultrasound for 20 s. The distance from the radial styloid process of the radial bone was noted, and the subsequent measurements of the radial artery after PO-FMD were performed in the same position. Then, a blood pressure cuff was placed on the left upper arm above the antecubital fossa. The upper arm cuff was inflated to a pressure 30 mmHg higher than the patient’s baseline systolic blood pressure for 10 min afterward, after which it was deflated, and the radial artery was imaged in the previous way (Fig. 2). The radial artery interface was continuously recorded by ultrasound for 5 min.

### Data analyses

**Figure 3:**
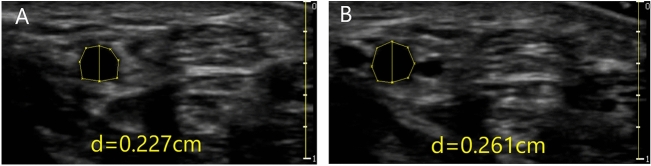
The measurement of radial artery diameter. (**A**) The baseline radial artery diameter. (**B**) The maximum radial artery diameter after PO-FMD. d = diameter (cm).

In this study, the sequences of intravascular ultrasound images were recorded in MP4 format at 24 frames/s and then converted to JPG format. The JPG files were then analyzed offline with a personal computer, using Digimizer software (MedCalc Software, Bluesnap, Belgium). The Digimizer software makes it possible to measure the changes in the vessels’ diameter in a chosen region of the ultrasound images as shown in Fig. 3. The measurements of each radial artery diameter were randomly by a single observer blinded to the study phase. The radial artery diameter was defined as the distance between the near wall and the leading edge of the lumen–intima interface of the far wall of the artery along a line perpendicular to the artery’s long axis using an electronic caliper. The baseline radial artery diameter, the maximum radial artery diameter, the time point at which the radial artery diameter is expanded to the maximum, and the time of radial artery dilation in the two groups were recorded.

### Statistical analysis

We used SPSS statistical software 26.0 to analyze all the statistical data. The Shapiro–Wilk normality test was used to compare distributions of data. For inter-group comparison, Students t-test was used for continuous normally distributed variables, while the Mann–Whitney U test was used for continuous non-normally distributed variables. The count data were compared by χ^2^ tests. Continuous and categorical variables are expressed as the Mean ± SD, median (interquartile range [IQR]), or frequency (%).

## Results

Over a four-month period, a total of 141 patients were recruited in the study. Thirty-nine of these patients were excluded based on the stated exclusion criteria summarized.

Ultimately, the remaining one hundred and two patients completed the study. The baseline characteristics and baseline radial artery diameter were well-matched among the two groups in Table 1. Higher rates of total cholesterol, low-density lipoprotein, hypertension, and diabetes mellitus were noted in the high-risk group. Otherwise, there were no statistically significant differences between the two groups.

**Table 1:** Patient characteristics and baseline radial artery diameter.

	LR (n = 51)	HR (n = 51)	*P* value
Age, year	47.49 ± 11.34	51.05 ± 10.82	0.107
Gender (M/F)	21/30	23/28	0.689
Body mass index	25.02 ± 3.35	25.36 ± 3.91	0.641
Creatinine, µmol/L	63.94 ± 11.19	66.88 ± 20.54	0.372
TG, mmol/L	1.45 ± 0.58	1.48 ± 0.56	0.816
TC, mmol/L	4.73 ± 0.82	5.46 ± 1.15	< 0.01
HDL, mmol/L	1.20 ± 0.26	1.12 ± 0.24	0.11
LDL, mmol/L	2.69 ± 0.67	3.42 ± 0.77	< 0.01
Hypertension	3 (5.8%)	20 (39.2%)	< 0.01
Diabetes mellitus	6 (11.8%)	17 (33.3%)	0.009
Dyslipidemias	5 (9.8%)	14 (27.5%)	0.013
Smoking	2 (3.9%)	6 (11.8%)	0.141
Chronic kidney diseases	0	0	NA
baseline radial artery diameter, mm	0.23 ± 0.05	0.24 ± 0.05	0.165

TG, triglycerides; TC, total cholesterol; HDL, high-density lipoprotein; LDL, low-density lipoprotein Data are presented as mean (standard deviation, SD) or n (proportion). LR, low-risk group; HR, high-risk group.

**Figure 4:**
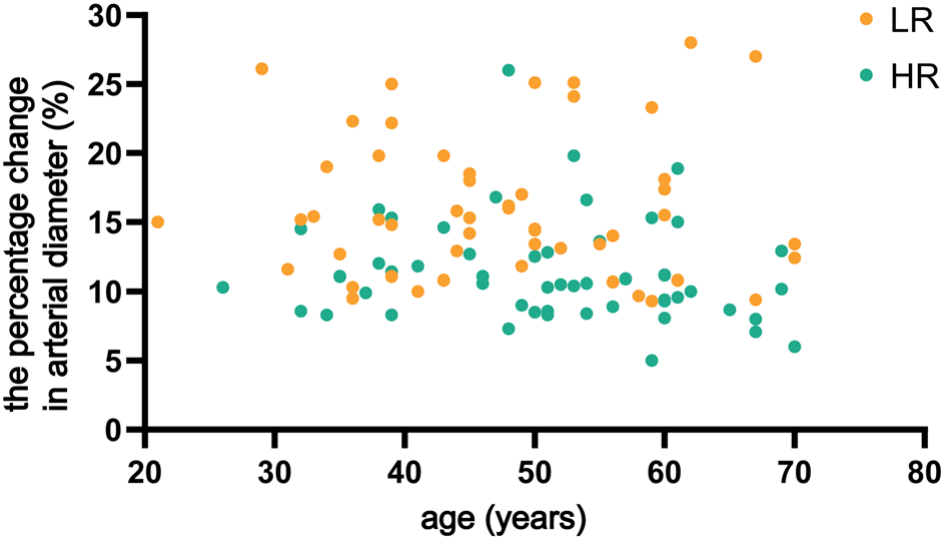
The relationship between the percentage change in arterial diameter and age for the LR group and HR group (scatterplots of the raw data). LR, low-risk group; HR, high-risk group.

The percentage change in arterial diameter in HR group was lower than LR group (median [interquartile range] 10.5 [8.6, 12.9] vs. 15.2 [12.4, 19.0] %). Taking into account the correlation between the percentage change in arterial diameter and age might be helpful in differentiating these two groups of patients. The scatterplots of the relationship between the percentage change in arterial diameter and age for the two groups are provided in Fig. 4.

**Figure 5:**
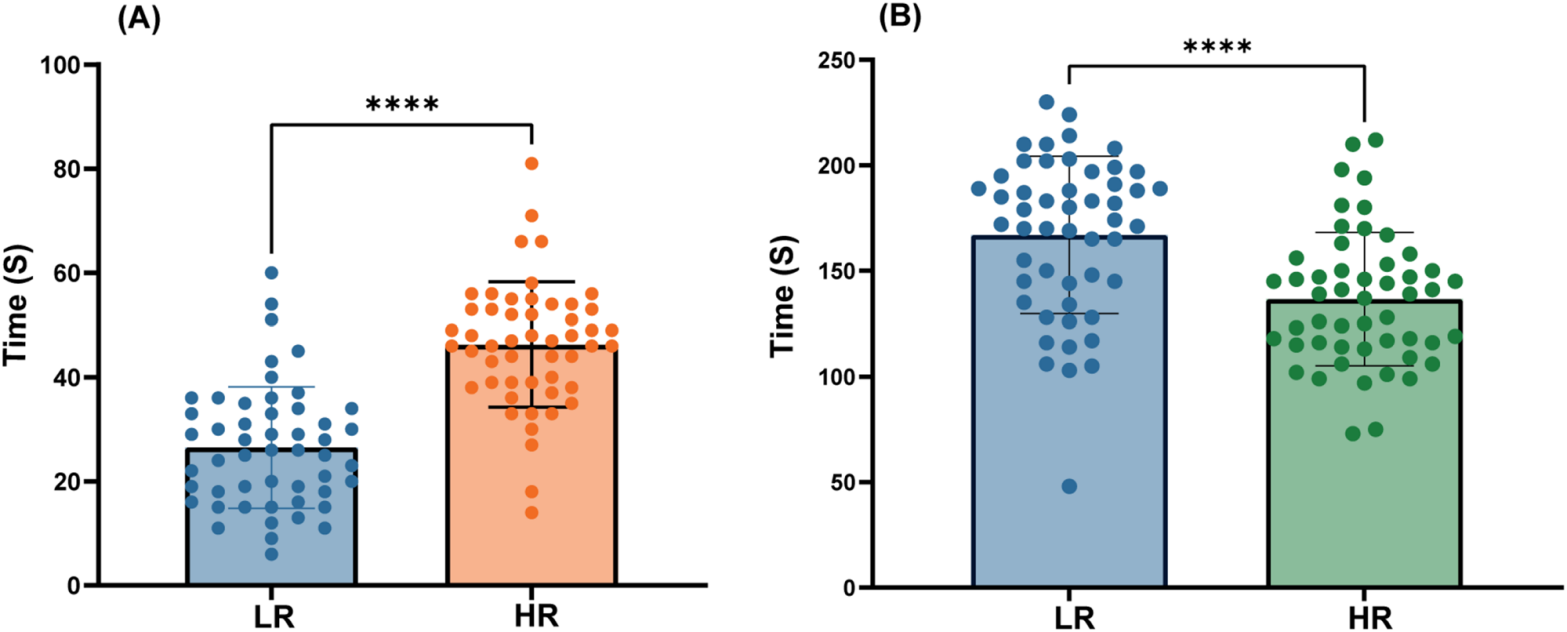
(**A**) Primary endpoint results: the time point at which the radial artery diameter is expanded to the maximum in the LR group and HR group. (**B**) Secondary outcomes: the time of radial artery dilation in the LR group and HR group. ****Represents* P* < 0.001. Bars represent mean ± standard deviation.

In regards to the primary endpoint, the time point at which the radial artery diameter is expanded to the maximum in the LR group and HR group was 26.49 ± 11.69 s and 46.27 ± 12.03 s, respectively (*P* < 0.001). The raw primary endpoint data are shown as scatterplots in Fig. 5(A). In regards to the secondary outcomes, the time of radial artery dilation in the HR group was significantly shorter than the LR group (mean [mean ± standard]: 136.65 ± 31.55 s vs. 168.98 ± 33.27 s). Figure 5(B) shows the scatterplots of the raw secondary outcomes data.

## Discussions

According to the available research, this present study was the first clinical pilot study to evaluate the duration and degree of radial artery dilatation induced after PO-FMD. In our study, the time point at which the radial artery diameter is expanded to the maximum in the LR group and HR group was 26 s and 46 s. Recent studies showed the time to peak (from cuff deflation to peak diameter) in patients with Type 2 Diabetes after FMD was 47 ± 13 s^12^. Broadly, these findings were similar to our results. By quantifying the time point of radial artery dilatation after PO-FMD, this will make clinical implementation of the adjunctive technology much more feasible. The results of our study indicate that the time of radial artery dilation and the percentage change in arterial diameter after PO-FMD decreased in high-risk groups compared to low-risk groups. And the time point at which the radial artery diameter is expanded to the maximum was delayed in high-risk groups. This might be associated with the impairment of endothelial function. There are multiple studies have demonstrated the association between impaired endothelial function and increased risk for atherosclerosis^13–15^, the impaired endothelial cells produce a weakened vasodilatation response to physiological or pharmacological stimulus (such as shear stress or acetylcholine infusion). Obviously, the results of our study were in line with these previous studies.

According to previous literature reports, the first-attempt success rate of radial artery catheterization with the pulse palpation technique ranges from 15 to 56% in adults^16^. The difficulty in radial artery catheterization is correlated with female gender, diabetes mellitus, hypertension, and dyslipidemia^17^. Females with small radial artery diameters may lead to more puncture attempts. The reason of patients with risk factors for cardiovascular diseases have a lower success rate of radial artery catheterization is not thoroughly studied. Possibly, this is due to the surrounding tissue trauma causing spasms or underlying atherosclerosis, causing these vessels to be more difficult to catheterize. Our study showed the percentage change in arterial diameter after PO-FMD was 10.5% (8.6–12.9%), and the duration of radial artery dilation was 136.65 ± 31.55 (s) in high-risk groups. Ying et al.^18^ found that FMD could relieve radial artery spasm which happens during puncture, and decreased puncture attempts after an initial failed attempt. These indicated the advantages of PO-FMD in patients with high risk factors for cardiovascular disease.

With the rapid development of visualization techniques, ultrasound has been increasingly used during vascular catheterization, such as deep venous punctures and radial artery punctures^19^. There are some studies that demonstrated the ultrasound-guided catheterization is successful more frequently than the pulse palpation method^20,21^. But the first attempt success rate and the time to establish the arterial line is much more operator dependent, and there is a learning curve to become proficient in this technique^12^. It also takes time to explore the vessels with ultrasound before radial artery puncture, and there are no anesthesiology-specific ultrasound machines in some township and county hospitals. However, PO-FMD needs no specialized equipment, and the cuff inflation can begin before the patients enter the operating rooms, making it possible to start procedures sooner. We foresee this technique will become a feasible option in busy operating rooms.

## Limitations

There are some limitations in this study. First, we studied only those patients awaking from general anesthesia are willing to consent to our studies, and some selection bias may be present. Second, the patients in the middle-risk group were excluded from this study, so the included patients were not comprehensive. Third, the sample size was relatively small, and each group consisted of 51 patients. Therefore, further studies with a large sample size are still needed to confirm the clinical significance of these preliminary results.

## Conclusions

In this study, the time and degree of radial artery dilatation induced after PO-FMD was evaluated. Moreover, the optimal puncture time point of PO-FMD in the LR group was 26 s, and in the HR group was 46 s. It will be easier to implement the adjunctive technology in the clinic by quantifying the degree and time of dilatation of the radial artery. It should be helpful to guide the time point in radial artery catheterization after PO-FMD. Future studies are needed to determine if this optimal puncture time is associated with an increased first-attempt success rate and reduced complications in radial artery catheterization. And this technique is easy, cheap, safe, and requires little specialized training or equipment. We foresee the most significant benefit of using PO-FMD in patients at risk for cardiovascular disease.

## Data Availability

The datasets generated during and/or analysed during the current study are available from the corresponding author on reasonable request.
